# Assessment of pelvic organ prolapse with the Pelvic Inclination Correction System: defining the normal range and threshold to pathology

**DOI:** 10.1007/s00261-024-04222-x

**Published:** 2024-03-13

**Authors:** Soleen Ghafoor, Stephan Beintner-Skawran, Cornelia Betschart, Thomas Winklehner, Cäcilia S. Reiner

**Affiliations:** 1 Institute of Diagnostic and Interventional Radiology, University Hospital Zurich, Raemistrasse 100, 8091 Zurich, Switzerland; 2 University of Zurich, Zurich, Switzerland; 3 Department of Nuclear Medicine, University Hospital Zurich, Raemistrasse 100, 8091 Zurich, Switzerland; 4 Department of Gynecology, University Hospital Zurich, Frauenklinikstrasse 10, 8091 Zurich, Switzerland; 5 Departments of Computer Graphics and Human-Computer Interaction, ARTORG Center for Biomedical Engineering Research, University of Bern, Berne, Switzerland

**Keywords:** Pelvic organ prolapse, Magnetic resonance imaging, Diagnostic imaging, Pelvic floor disorders

## Abstract

**Purpose:**

To define the normal range and threshold values for pathologic prolapse on MRI using the PICS line and assess its correlation with the pubococcygeal line (PCL).

**Methods:**

This prospective, IRB-approved study included 20 nulliparous volunteers and 18 prolapse patients (POP-Q Stage ≥ 2). Organ positions (bladder, cervix, anorectal junction) relative to PICS and PCL were measured on dynamic MRI. Differences in organ position were compared. Receiver-operating characteristic (ROC) analysis was performed to identify cutoff values for prolapse using the PICS line. The correlation between PICS and PCL measurements was tested with Spearman’s rank correlation.

**Results:**

In volunteers, median bladder and cervix positions measured to the PICS at rest were − 2.7 cm and − 5.3 cm compared to − 1.9 cm and − 2.7 cm in patients (*p* < 0.001). During straining, bladder and cervix were at − 0.9 cm and − 3.2 cm in volunteers versus + 2.5 cm and + 2.5 cm in patients (*p* < 0.001). Correlation was strong for PICS and PCL measurements for all three compartments (*δ* = 0.883–0.970, *p* ≤ 0.001). AUCs of PICS for the anterior and middle compartment were 0.98 (95% confidence interval [CI] 0.96–1.00, *p* < 0.001) and 0.96 (95% CI 0.89–1.00, *p* < 0.001) for differentiating patients from healthy volunteers. AUC for the posterior compartment was 0.76 (95% CI 0.57–0.96, *p* = 0.034).

**Conclusion:**

PICS measurements reliably differentiate patients from volunteers in the anterior and middle compartment. Future studies need to identify a reliable threshold for the posterior compartment. PICS and PCL measurements are strongly correlated.

**Graphical abstract:**

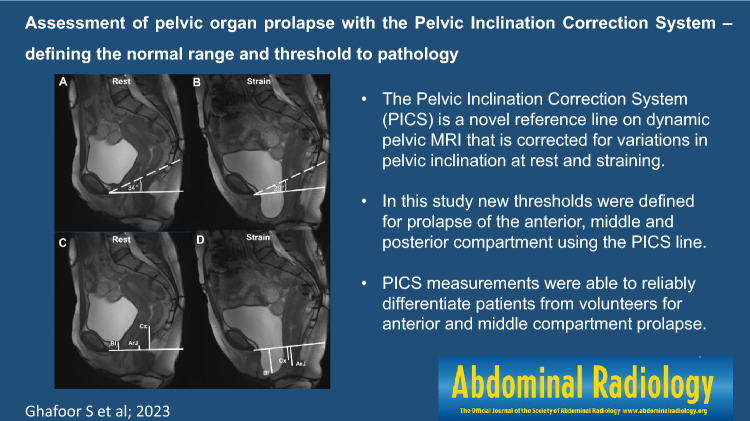

## Introduction

Pelvic organ prolapse (POP) defines the descent of pelvic organs from their normal position along the longitudinal body axis in a gravity-dependent manner. It affects many women with prevalence rates of up to 50% [1]. Risk factors include age, vaginal delivery, and higher body mass index. A variety of symptoms are associated with POP, including a sensation of pelvic pressure, lower abdominal discomfort, voiding dysfunction, issues with defecation, and impaired sexual function [2].

Physical examination is an essential cornerstone in the diagnosis of POP. Over the last years, magnetic resonance imaging (MRI) has become increasingly important as an invaluable supplemental tool in assessing pelvic floor disorders. Its exceptional soft tissue resolution and ability to capture dynamic images offer precise details about pelvic floor morphology and anatomy [3]. Dynamic MRI of the pelvic floor includes image acquisition at rest and straining and enables grading severity of POP through measurements of organ positions at rest and straining using reference lines [3]. Different pelvic reference lines are discussed in the literature and applied in clinical practice, including frequently used ones such as the pubococcygeal line (PCL) and midpubic line (MPL) [4–6]. The existing reference lines are not corrected for the pelvic inclination at rest or straining which could introduce a systematic error in measurements, lead to variation between measurements and affect standardization of quantification [5]. This assumes particular significance in circumstances where MRI measurements are integrated into composite endpoints for patients undergoing treatment for POP, necessitating a high degree of standardization and reproducibility [7]. Furthermore, the correlation of MRI-based measurements using existing reference lines with clinical POP grading and symptom severity is moderate at best [8–10]. The Pelvic Inclination Correction System (PICS) was proposed as a new reference line [5, 11]. The PICS is based on a fixed clockwise rotation with respect to the sacrococcygeal–inferior pubic point (SCIPP) line. Given the near-horizontal direction of the PICS line, organ point measurements are nearly independent of the antero-posterior organ location, whereas measurements using other more oblique reference lines could be affected by the baseline organ point position in the antero-posterior direction [5]. Further, this reference system is independent of pelvic position within the MRI scanner as it corrects for variations in pelvic inclination at rest and straining [12]. These variations of pelvic inclination were previously described and could influence the position of the bony landmarks that some reference lines are based on [5].

However, as opposed to other reference lines, the normal range and threshold values to pathology have not yet been defined for the PICS line, limiting its utility in daily clinical practice and its comparison to other existing reference lines.

Therefore, the purpose of this study was to define the range of normal organ positions in relation to the PICS line and to identify specific threshold values for pathologic pelvic organ descent using a cohort of volunteers and symptomatic prolapse patients. In addition, PICS measurements were compared to established PCL measurements.

## Materials and methods

### Patients and volunteers

This prospective study was approved by the local ethics committee (Study-ID: BASEC 2018-01107), and all patients and volunteers gave written informed consent.

Twenty-two consecutive volunteers and 25 consecutive patients from the urogynecology unit of our hospital were enrolled.

Patients were included, if they had symptoms of POP as assessed with a standardized questionnaire [13] and a stage 2 or more prolapse in any compartment as assessed through urogynecologic examination using the clinical “Pelvic Organ Prolapse Quantification (POP-Q)” system [14]. The POP-Q system ranges from 0 (no prolapse) to 4 (maximum descent) scored according to the extent of organ prolapse relative to the hymen as the anatomic reference point [15].

The standardized and validated pelvic floor questionnaire that was used [13] integrates four domains of pelvic floor dysfunction (bladder function, bowel function, sexual function, and pelvic organ prolapse), grades their severity, and assesses bothersomeness and condition-specific quality of life. For each of the four domains, a total value will be returned and calculated into a total “pelvic floor dysfunction score” which can reach a maximum of 40 points. A higher score correlates with increased severity of pelvic floor dysfunction.

Volunteers were included if symptoms of pelvic organ prolapse were absent (assessed through a structured interview prior to inclusion and with the standardized questionnaire) and if they had never given birth (nulliparity).

Additional inclusion criteria comprised obtaining written informed consent and the complete MRI exam according to study protocol. Exclusion criteria were inability to follow the instructions during the MR image acquisition, failure to return the questionnaire or undergo physical examination (for patients only), history of prior pelvic floor surgery, insufficient straining maneuver during the dynamic phase of the MRI, and general contraindications to MRI (e.g., presence of non-MR-compatible metallic implants, devices or metallic foreign bodies). A part of this cohort was previously used for another study with a different research objective that focused on comparison of different techniques for acquisition of dynamic MRI sequences [16].

### MRI protocol

MRI examinations were performed on a 3.0 T clinical MRI scanner (Skyra, Siemens Healthineers, Erlangen, Germany) with a 60-channel array coil. Subjects were examined in supine body position and emptied their bladder 15 min prior to the exam. Prior to image acquisition, the participants were instructed by the MR technologist on how to perform the straining maneuver for the dynamic phases of the examination and were allowed to practice the maneuver before the image acquisition. In this study, we analyzed the dynamic midsagittal single-slice sequences obtained at rest and straining (true fast imaging with steady state free precession [TRUFI], TR/TE, 460/1.5 ms; matrix, 320 × 320; FOV, 240 × 240 mm; slice thickness, 10 mm). Images were acquired during three consecutive straining maneuvers (total acquisition time 1 min 10 s, 72 consecutive images per straining maneuver).

### Image analysis

**Fig. 1:**
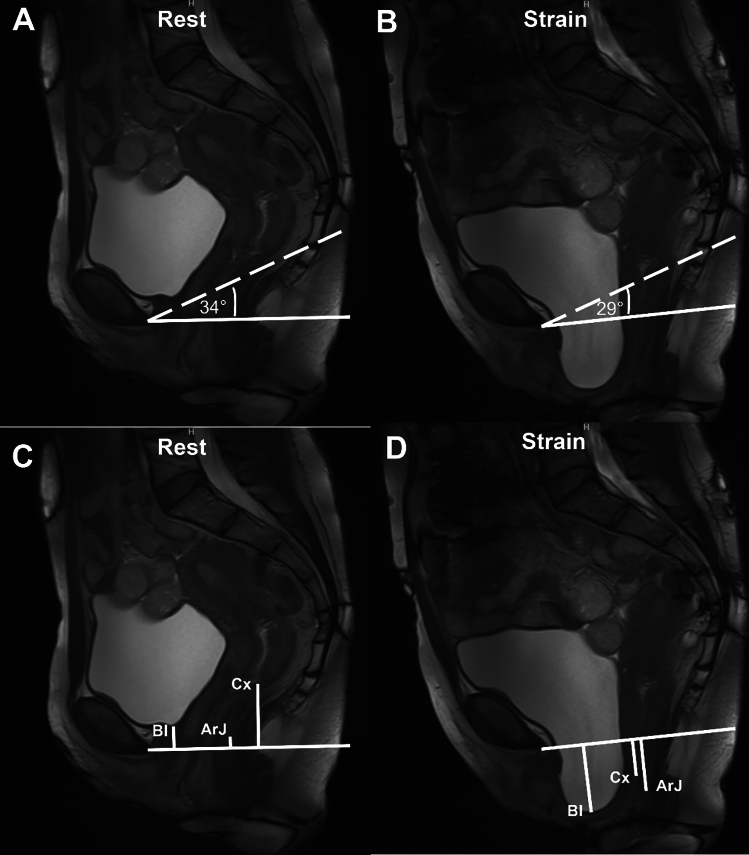
38-year-old (gravida 2, para 2) patient with multi-compartment organ prolapse presenting with symptoms of vaginal bulge and stress urinary incontinence. Top row depicts dynamic midsagittal MR images showing the position of the PICS line at rest (**A**) and straining (**B**) adjusted for differences in pelvic inclination. The interrupted line represents the sacrococcygeal-inferior pubic point (SCIPP) line which connects the sacrococcygeal joint to the inferior border of the pubic symphysis. The PICS line (solid line in **A**–**D**) is then drawn from the inferior border of the pubic symphysis with a 34° and 29° clockwise rotation from the SCIPP line at rest and straining, respectively. Bottom row (**C**, **D**) shows organ point measurements with reference to the PICS line in this patient at rest (**C**) and straining (**D**). At rest, the bladder (Bl), cervix (Cx), and anorectal junction (ArJ) are located at 1.5 cm, 4.2 cm, and 0.9 cm above the PICS line. At straining, the bladder descents to 4.0 cm below, the cervix to 1.9 cm below, and the anorectal junction to 3.1 cm below the PICS line. Measurements are made perpendicularly to the PICS line. Clinically, the patient was graded as POP-Q III° for the anterior, POP-Q II° for the middle, and POP-Q I° for the posterior compartment

One radiologist (***S.G.***, 7 years of experience in abdominal imaging) annotated the organ points of the bladder (anterior compartment), cervix (middle compartment), and anorectal junction (posterior compartment) at rest and straining in volunteers and patients. The straining phase depicting the maximum pelvic organ descent was used for measurements. Annotations were done on an in-house developed tool called “3D PICS” [11]. Briefly, this tool is based on a 3D coordinate system using pre-defined bony landmarks (the inferior margin of the symphysis, the sacrococcygeal joint, and the ischial spines) and uses the PICS plane as a reference line. The PICS plane is drawn as a line with 34 degrees (for images at rest) or 29 degrees (for images at straining) clockwise rotation from the “sacrococcygeal inferior—pubic point line” (SCIPP line) (Fig. 1). The SCIPP line is a line connecting the inferior margin of the symphysis to the anterior sacrococcygeal joint. The need for adjustments in rotational angles (i.e., 34 and 29 degrees) between images at rest and straining is informed by earlier research demonstrating variations in pelvic tilt between these states [5]. To account for these differences and ensure accurate measurements, the line is modified accordingly depending on whether measurements are made on resting or straining images. The coordinates of any given point annotated on MR images can be calculated using 3D PICS. When using single-slice midsagittal TRUFI images, two coordinates are calculated (position along the y-axis in the craniocaudal direction, and the position along the x-axis in the antero-posterior direction) [11]. For better comparison with the PCL, we analyzed the position in the y-axis (craniocaudal direction) only. The PCL represents a straight line connecting the inferior border of the pubic symphysis to the last coccygeal joint and is the most widely used reference line for the grading of POP on MRI [3].

The 3D PICS tool is based on organ point locations measured perpendicularly to the PICS line. For the manual measurements of the organ point positions relative to the PCL, the measurements were also done perpendicularly to the PCL [3]. For the PCL and PICS measurements alike, coordinates of organ points located above the reference line are labeled with a negative sign and those below the reference line with a positive sign (Fig. 1).

In addition, images were reviewed in the Picture communication and archiving system and the position of the three organ points was measured at rest and straining in reference to the PCL. To define prolapse based on PCL measurements, we used established thresholds that were published in the joint consensus recommendations of the ESUR and ESGAR Pelvic Floor Working Group [3].

### Statistical analysis

Categorical variables were described as frequencies and percentages. Continuous variables were described as means and standard deviations or medians and interquartile range where appropriate.

Demographics of the study population and organ point measurements at rest and straining in patients and volunteers were analyzed using descriptive statistics. Differences in age and body mass index between patients and volunteers were tested with the Mann–Whitney U test.

Pelvic organ point measurements were tested for normal distribution using the Kolmogorov–Smirnov test.

Differences in organ positions at rest and straining between patients and volunteers were tested with a student’s t-test or Mann–Whitney U test. In patients, differences in the proportion of different POP-Q stages for the anterior, middle, and posterior compartment were tested with a McNemar-Bowker test.

Receiver-operating characteristic (ROC) curves with calculation of the area-under-the-curve (AUC) were plotted to identify compartment-wise cutoff values for pelvic organ point measurements at straining for diagnosis of pathologic pelvic floor descent. The 95% confidence intervals (CI) were computed using a bootstrapping approach. Following thresholds were applied to assess the discriminative power using the AUC: < 0.5 = not useful, 0.5–0.6 = poor, 0.61–0.7 = acceptable, 0.71–0.8 = good, 0.81–0.9 = very good, and 0.91–1.0 = excellent [17]. Cutoff values for organ point positions from the ROC analysis were set to yield high sensitivity and specificity for the differentiation between patients (with POP-Q stage ≥ 2 in the respective compartment) and healthy volunteers. In addition, the sensitivity and specificity of PCL measurements for differentiation between patients and volunteers were calculated using established threshold values for the PCL at straining [3].

The correlation between PICS measurements and PCL measurements and compartment-wise POP-Q stage was tested with Spearman’s rank correlation. The Spearman’s coefficient (*δ*) ranges from − 1 to + 1, where 0 indicates that there is no linear association and 1 indicates perfect linear association [18]. The following thresholds were applied to assess the strength of relationship: < 0.2 = no or negligible relationship, 0.20–0.29 = weak relationship, 0.30–0.39 = moderate relationship, 0.40–0.69 = strong relationship, and ≥ 0.70 = very strong relationship [19]. Differences in sensitivity and specificity between PICS measurements and PCL measurements were compared with a McNemar test.

A 2-tailed *p* value of < 0.05 was used to determine the statistical significance. Statistical analyses were performed with SPSS (version 29, IBM Corporation, Armonk, NY, USA).

## Results

### Patient and volunteer characteristics

Two volunteers and seven patients were excluded. Reasons for exclusion were suboptimal straining effort (volunteer, *n* = 2; patient, *n* = 5), failure to undergo physical exam (patient, *n* = 1), and incomplete MRI exam due to inability to follow the instructions during image acquisition (patient, *n* = 1). The final study population comprised 20 volunteers and 18 patients. Baseline characteristics of the patients and volunteers are depicted in Table 1.

**Table 1:** Baseline characteristics of patients and volunteers

	Patients (*n* = 18)	Volunteers (*n* = 20)
Age (years)	39.4 ± 4.7	24.3 ± 3.9
BMI (kg/m2)	21.9 ± 3.3	21.6 ± 2.9
Parity (count)	2 (1–3)	N/A
Maximum birth weight (g)*	3985 (3475–4263)	N/A
POP-Q stage		N/A
Anterior compartment		
Grade 0	0	
Grade 1	2/18 (11.1%)	
Grade 2	14/18 (77.8%)	
Grade 3	2/18 (11.1%)	
Grade 4	0	
Middle compartment		
Grade 0	3/18 (16.7%)	
Grade 1	8/18 (44.4%)	
Grade 2	5/18 (27.8%)	
Grade 3	2/18 (11.1%)	
Grade 4	0	
Posterior compartment		
Grade 0	6/18 (33.3%)	
Grade 1	10/18 (55.5%)	
Grade 2	2/18 (22.2%)	
Grade 3	0	
Grade 4	0	
Pelvic floor questionnaire		
Total score	8.7 (6.8, 11.5)	1.6 (0.3, 2.5)
Sub-score: bladder function	1.7 (0.8, 2.7)	0.3 (0.0, 0.4)
Sub-score: bowel function	1.9 (1.2, 3.3)	1.0 (0.3, 1.5)
Sub-score: prolapse	3.3 (2.2, 6.0)	0.0 (0.0, 0.0)
Sub-score: sexual function	1.7 (0.2, 2.7)	0.0 (0.0, 0.1)

Data are presented as either mean ± standard deviation, numbers (percentage), or median (range or interquartile range)

*BMI* body mass index, *POP-Q* Pelvic Organ Prolapse Quantification System

*Maximum weight of vaginally delivered baby

A POP-Q stage ≥ 2 prolapse of the anterior compartment was present in 16 patients (88.9%), of the middle compartment in 7 patients (38.9%), and of the posterior compartment in 4 patients (22.2%). There was a statistically significant difference in the proportion of higher POP-Q stages in the anterior compartment compared with the middle compartment (*p* = 0.004) and posterior compartment (*p* = 0.003).

### Organ point measurements in patients and healthy volunteers

Median organ point positions in reference to the PICS line at rest and straining are depicted in Table 2*.*

**Table 2:** Organ point measurements in volunteers and patients at rest and straining

	Organ position* (cm)	
	Patients (POP-Q ≥ 2)	Volunteers
Rest		
Anterior compartment	**− 1.9** (− 2.2, − 1.4)	**− 2.7** (− 2.9, − 2.5)
Middle compartment	**− 2.7** (− 3.4, − 2.4)	**− 5.3** (− 5.6, − 4.6)
Posterior compartment	**− 2.2** (− 2.9, − 1.2)	**− 2.9** (− 3.0, − 2.6)
Straining		
Anterior compartment	** + 2.5** (+ 1.7, + 3.1)	**− 0.9** (− 1.7, − 0.2)
Middle compartment	** + 2.5** (+ 0.5, + 3.9)	**− 3.2** (− 3.9, − 1.8)
Posterior compartment	** + 0.4** (+ 0.1, + 1.3)	**− 0.5** (− 2.0, + 0.7)

Data are presented as medians (numbers in bold) and interquartile range (numbers in parentheses)

*Organ position along the craniocaudal direction (*y*-axis) in reference to the PICS plane. Negative values are located above and positive values below the PICS plane

The positions of bladder and cervix were significantly lower in patients than in volunteers at rest (*p* < 0.001, *p* < 0.001) and at straining (*p* < 0.001, *p* < 0.001). There were no statistically significant differences in the position of the anorectal junction in patients and volunteers at rest and straining (*p* = 0.353, *p* = 0.157). 

**Fig. 2:**
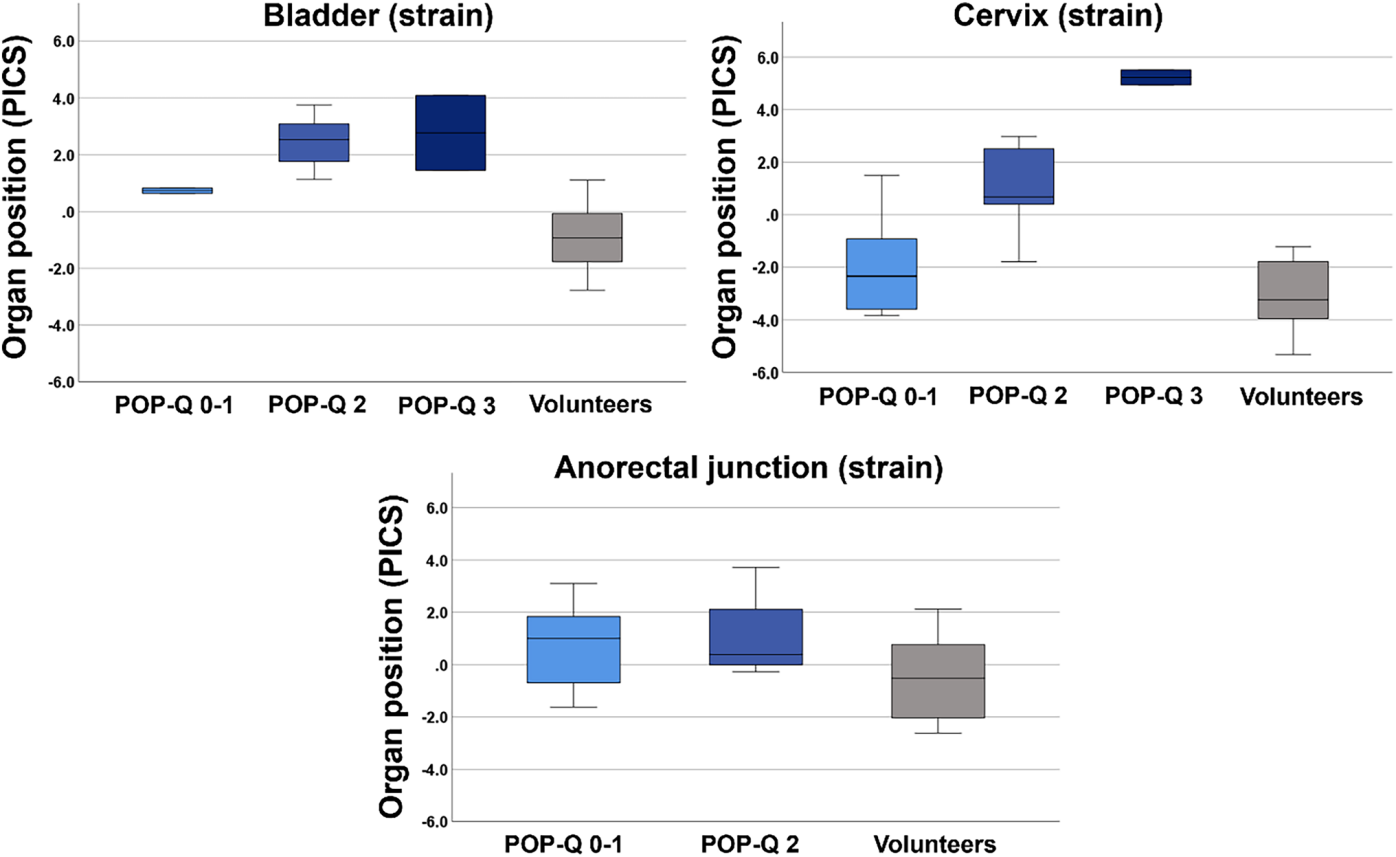
Boxplots depicting the position of the bladder, cervix, and anorectal junction at straining with reference to the PICS line in patients with different POP-Q stages and in volunteers

The median organ positions in patients according to POP-Q stages and the organ positions in volunteers are depicted in Fig. 2**.**

### Correlation of PICS and PCL

**Fig. 3:**
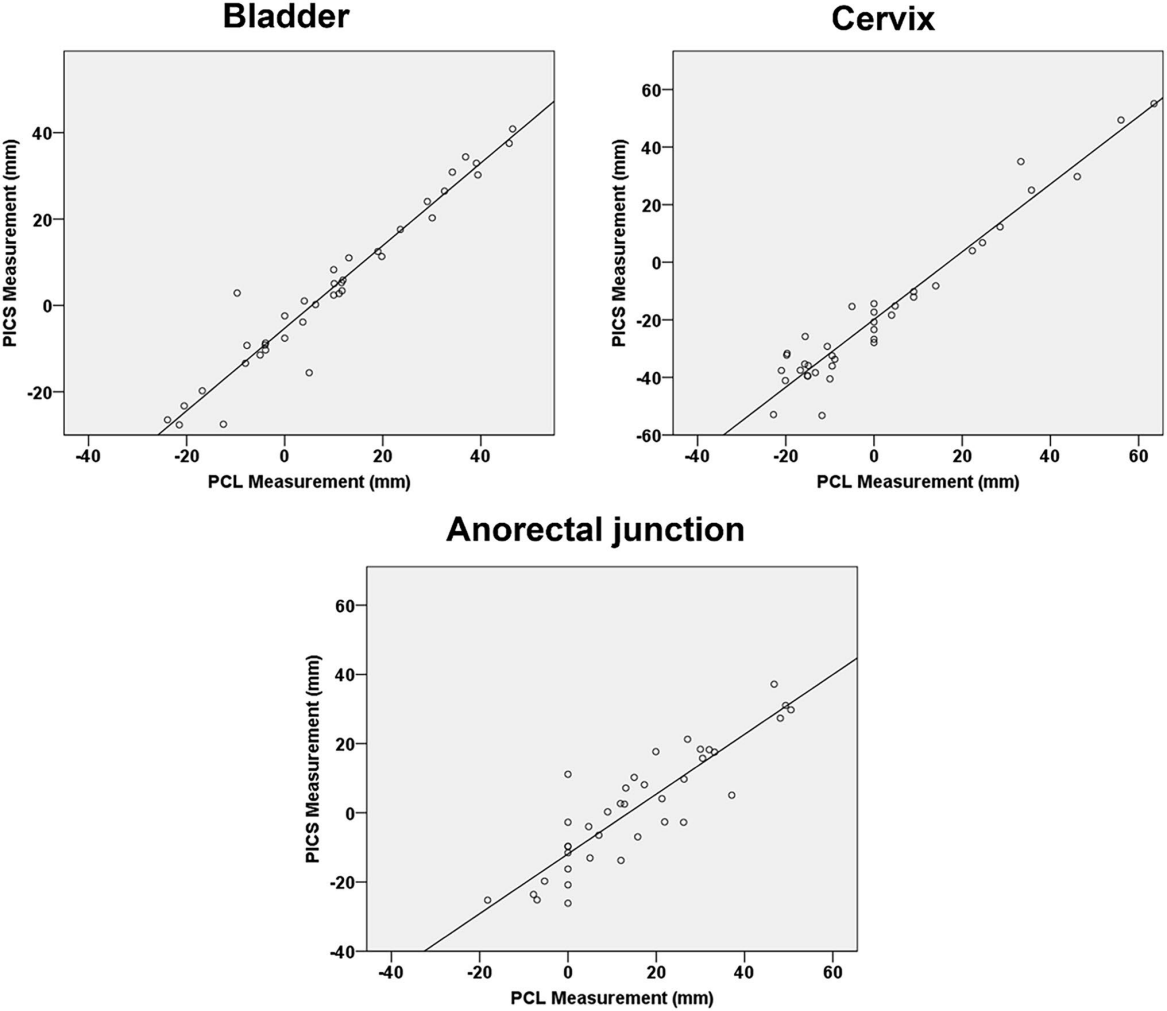
Scatterplots and linear regression lines showing the correlation between PICS measurements (*X*-axis) and PCL measurements (*Y*-axis) for the bladder (*δ* = 0.970, *p* ≤ 0.001), cervix (*δ* = 0.969, *p* ≤ 0.001), and anorectal junction (*δ* = 0.883, *p* ≤ 0.001)

The PICS measurements along the y-axis strongly correlated with the PCL measurements for the anterior compartment (*δ* = 0.970, *p* ≤ 0.001), middle compartment (*δ* = 0.969, *p* ≤ 0.001), and posterior compartment (*δ* = 0.883, *p* ≤ 0.001) (Fig. 3*)*.

### Potential PICS threshold and diagnostic performance for POP at straining

**Fig. 4:**
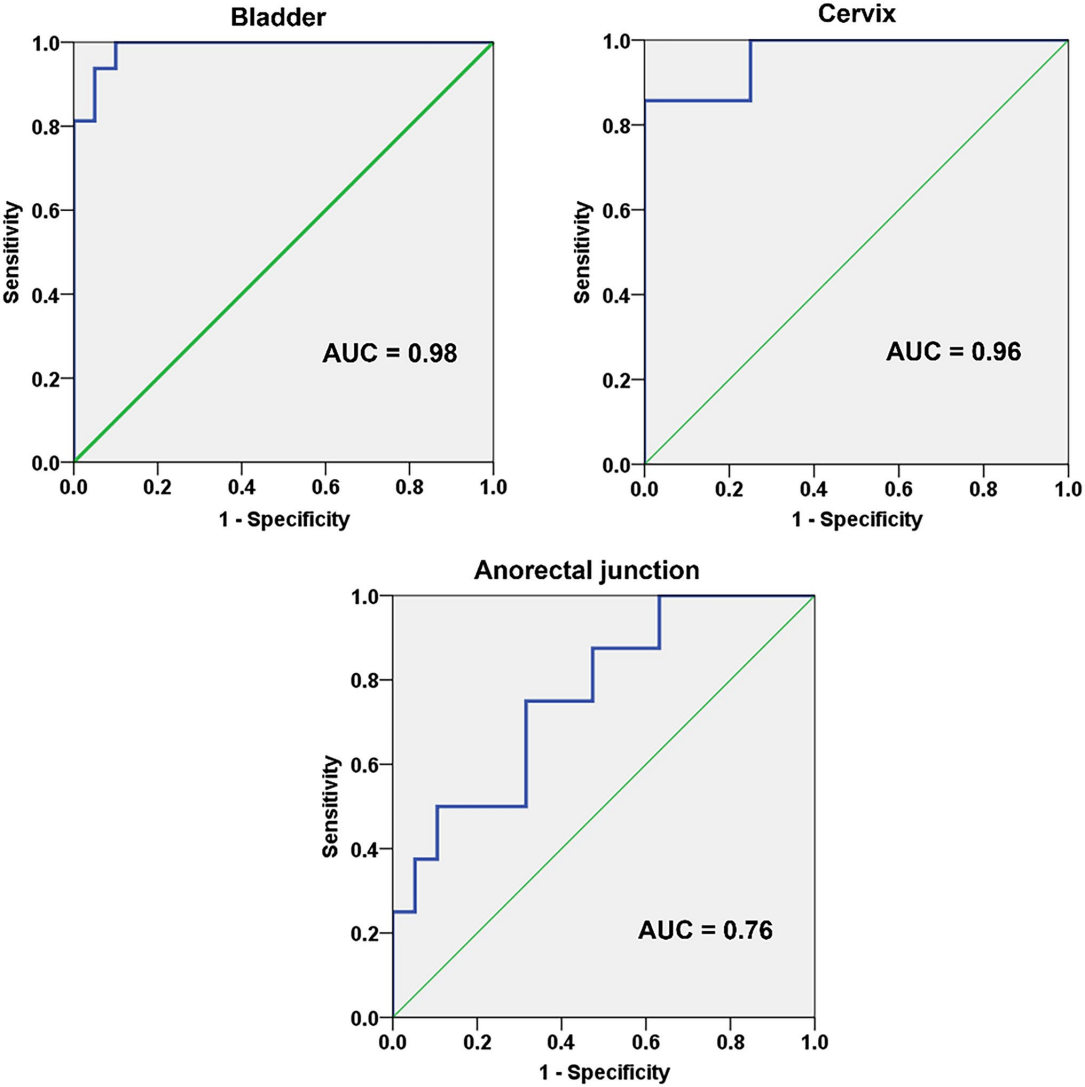
ROC analysis of PICS measurements for the anterior compartment (bladder), middle compartment (cervix), and posterior compartment (anorectal junction)

The ROC analysis of measurements for the anterior compartment revealed an AUC of 0.98 (95% CI 0.96–1.00, *p* < 0.001) for differentiating patients (with POP-Q stage ≥ 2) from volunteers. At a cutoff value of 0.5 cm below the PICS line, the sensitivity was 100% and specificity was 90%. Using this cutoff, all patients with a POP-Q ≥ 2 would have been correctly identified and only one volunteer misclassified. None of the volunteers had a bladder position at straining that was lower than 1.1 cm below the PICS plane (Fig. 4).

The ROC analysis of measurements for the middle compartment revealed an AUC of 0.96 (95% CI 0.89–1.00, *p* < 0.001) for differentiating patients (with POP-Q stage ≥ 2) from volunteers. At a cutoff value of 0.4 cm above the PICS plane, the sensitivity was 86% and specificity was 100%. The lowest position of the cervix at straining among the volunteers was 1.2 cm above the PICS plane (Fig. 4).

The ROC analysis of measurements for the posterior compartment revealed an AUC of 0.76 (95% CI 0.57–0.96, *p* = 0.034) for differentiating patients (with POP-Q stage ≥ 2) from volunteers. At a cutoff value of 0.2 cm below the PICS plane, the sensitivity was 75% and specificity was 68%. The lowest position of the anorectal junction among the volunteers was 2.1 cm below the PICS plane and the highest position among the patients was at the level of the PICS plane (Fig. 4).

### Diagnostic performance of the PCL threshold for POP at straining

When using the established PCL threshold values for pathologic organ descent at straining [3] for the anterior (> 1 cm below the PCL), middle (> 1 cm below the PCL), and posterior compartment (≥ 3 cm below the PCL), the sensitivity and specificity were 100% and 82% for the anterior compartment, 86% and 93% for the middle compartment, and 63% and 87% for the posterior compartment.

### Correlation of PICS with POP-Q stage

There was a strong positive correlation between PICS measurements and POP-Q stage for the anterior compartment (*δ* = 0.578, *p* = 0.045) and middle compartment (*δ* = 0.655, *p* = 0.003) but not for the posterior compartment (*δ* = 0.245, *p* = 0.326).

#### Comparison of PICS and PCL

There were no statistically significant differences for the sensitivity (PICS: 100%, PCL: 100%) and specificity (PICS: 90%, PCL: 82%) between the PICS and PCL measurements for the anterior compartment (*p* = 1.000).

There were no statistically significant differences for the sensitivity (PICS: 86%, PCL: 86%) and specificity (PICS: 100%, PCL: 93%) between the PICS and PCL measurements for the middle compartment (*p* = 1.000).

There was a statistically significant difference in the sensitivity (PICS: 75%, PCL: 63%) and specificity (PICS: 68%, PCL: 87%) between the PICS and PCL measurements for the posterior compartment (*p* = 0.002).

## Discussion

This prospective study is the first to aim at defining the normal range and thresholds for pathologic organ descent at straining using the PICS line as a reference in two distinct populations: a urogynecologic cohort of premenopausal women with pelvic organ prolapse and a reference population of nulliparous asymptomatic volunteers. Based on the results of ROC analysis, a cutoff of 0.5 cm below the PICS line at straining in the anterior compartment, and of 0.4 cm above the PICS line at straining in the middle compartment were identified as optimal for distinguishing between volunteers and patients. For ease of clinical application, the level of the PICS line itself (i.e., 0 cm) could be utilized as the threshold, whereby descent of the bladder or cervix beyond this point during straining would be classified as pathological. For the posterior compartment, the ROC analysis revealed a cutoff of 0.2 cm below the PICS line. However, as opposed to the excellent AUC for the anterior and middle compartment, the AUC for the posterior compartment was lower.

This study included a urogynecologic patient population with anterior and middle compartment-predominant symptoms which was reflected in the higher prevalence of POP-Q stage ≥ 2 anterior compartment descent (88.9%) compared with 38.9% and 22.2% for the middle and posterior compartment. Due to the low prevalence of posterior compartment prolapse in our cohort, the AUC for the posterior compartment was found to be worse in our population. Consequently, we recommend that the proposed threshold for the posterior compartment be subjected to further evaluation and refinement, and preferably tested in a cohort with symptomatic posterior compartment prolapse, to enhance their discriminatory power.

The diagnostic performance of the PICS line for the diagnosis of POP-Q ≥ 2 pelvic organ prolapse using our thresholds was high with an AUC of 0.98 and 0.96 for the anterior and middle compartment, respectively. In a study by Pannu et al. [20] using the PCL and MPL, the performance of MRI measurements to identify pathologic POP was lower with the highest agreement between MRI measurements and clinical examination for the anterior compartment (79% for the PCL and 85% for the MPL). Our study showed a strong positive correlation between PICS and PCL measurements. Furthermore, there were no statistically significant differences in sensitivity and specificity of the PICS compared with the established PCL for the diagnosis of POP in the anterior and middle compartment. These findings indicate that the PICS line could be used for assessment of organ prolapse as it performs similarly to the established PCL. However, the additional correction for changes in pelvic tilt inherent to the PICS line could be an advantage over the PCL, but future research is needed to explore this hypothesis and to investigate these PICS thresholds in larger and more diverse patient cohorts with pelvic organ prolapse.

Although the PICS line has been used in some studies since its inception [21–24], it has yet to be implemented in clinical practice. Furthermore, its use in research and comparison of its performance to other established lines has been limited by the absence of published threshold values for this line.

Using our study population, we have identified thresholds with excellent discriminatory power for the anterior and middle compartment. For the bladder, any measurements that are more than 0.5 cm below the PICS line during straining can be considered as pathological. For the cervix, any measurements lower than 0.4 cm above the PICS line during straining can be considered as pathological. A grading of POP based on PCL has been proposed in addition to defining a threshold to pathology [3]. However, it was not investigated to date whether the PCL-based grading of POP on MRI correlates with symptom severity or severity of clinical prolapse grade. Furthermore, it should be noted that MRI-based POP measurements using established reference lines do not necessarily correlate well with clinical symptoms and clinical prolapse grade [20, 25, 26]. For example, Fauconnier et al. [25] found that the PCL and perineal line showed good interreader agreement but were poorly correlated with clinical prolapse grades. Similarly, Cortes et al. [26] analyzed the agreement between prolapse grading through clinical examination and MRI measurements using the MPL in 51 women presenting with symptoms of prolapse and found only 41.1% agreement between clinical prolapse grade and MRI. Moreover, studies showed that clinical POP-Q stages themselves do not always correlate well with clinical symptoms either [27]. Therefore, after defining thresholds to pathology with the PICS line, future studies should focus on establishing thresholds for different grades of organ prolapse using the PICS line ideally matching PICS grading to clinical prolapse grade and symptom severity.

Rechi-Sierra et al. [9] compared three reference lines, namely PCL, MPL, and the H-line to physical examination and found low agreement between clinical prolapse stage and MRI grade using the MPL and PCL. The H-line is drawn from the inferior ramus of the pubic symphysis to the reflection of the puborectalis muscle in the posterior rectum. In that study, the H-line showed the highest agreement with clinical prolapse grade (kappa index of 0.602–0.618) [9]. However, the H-line has not yet been studied thoroughly for the diagnosis and grading of organ prolapse, rather it represents a measure for the assessment of pelvic floor relaxation [28]. Furthermore, since it is a dynamic line, its length depends on the degree of contraction or relaxation of the pelvic floor muscles. Although this may more closely simulate the clinical POP-Q system (where the soft tissue hymenal ring serves as a reference point), use of a moving soft tissue landmark introduces additional variability which may affect measurement reproducibility [5]. In addition, the puborectalis reflection may not always be very distinctly visible on all midsagittal MR images. These factors could affect the reproducibility of the H-line compared to reference lines that are based on bony landmarks.

Despite the existing limitations with regard to symptom correlation with clinical and MRI findings in pelvic organ prolapse, dynamic MRI of the pelvis is not only useful in the work-up of patients with pelvic floor disorders but could play an important role as part of a composite endpoint metric in patients treated for symptomatic pelvic organ prolapse in the future [29]. Composite endpoints often combine subjective and objective metrics to get a better picture of outcome, since single endpoints such as patient-reported symptoms can be subjective and variable. In this setting, dynamic MRI of the pelvis could be incorporated as part of such a composite endpoint since it allows for an objective and reproducible assessment of pelvic organ prolapse.

In this study, we found a strong positive correlation between PICS measurements and POP-Q stages for the anterior and middle compartment, but not for the posterior compartment. This is likely related to the low prevalence of posterior compartment pathology in our cohort and will need to be re-evaluated in the future using a cohort with a higher prevalence of posterior compartment prolapse.

Furthermore, studies have shown that a higher degree of prolapse is measured when obtaining a defecation phase compared to a maximum straining phase only [30]. Therefore, additional adapted thresholds for organ prolapse using the PICS line will need to be defined for the defecation phase on MRI in the future. A prior study by Schawkat et al. [31] found that applying the established PCL thresholds for the posterior compartment [3], which are based on the straining and not defecation phase, would lead to an overestimation of posterior compartment prolapse on MR defecography. To date, the PICS line is only corrected for changes of pelvic tilt between rest and straining, but not for the defecation phase, which induces a higher degree of stress on the pelvic floor and likely also affects pelvic tilt [5]. Therefore, future studies should also focus on adapting the PICS plane for the defecation phase.

Our study has several limitations. First, our population is relatively small. Nevertheless, we were able to define specific thresholds with good discriminatory power in this prospective explorative study, but our findings will need to be validated in larger and more diverse cohorts in the future (e.g., in postmenopausal volunteers without symptomatic prolapse). Second, our population had a low prevalence of higher-grade posterior compartment prolapse which had an impact on the results and obtained thresholds. Future studies should investigate patients with posterior compartment-predominant pathology to validate and refine the thresholds established in this study. This will improve discriminatory power. Our study only included premenopausal patients who were recruited according to a prospective study protocol. Pelvic organ prolapse is more frequent in postmenopausal women and different thresholds may apply to this population [32]. Last, our study was based on measurements taken during the straining phase and did not include a defecation phase. Previous studies have shown that the evacuation phase of dynamic MRI is more sensitive in identifying prolapse and can depict more severe grades of POP [30]. Therefore, the current thresholds for the PICS line may not apply to MR defecography and future studies will need to focus on establishing adapted PICS thresholds for diagnosis of POP for the defecation phase.

## Conclusion

A position of the bladder base or the cervix below the PICS line at straining indicates a pathological descent. PICS measurements allow differentiation of urogynecologic patients with POP-Q stage ≥ 2 in the anterior and middle compartment from asymptomatic volunteers. Future studies including more patients with posterior compartment pathology are needed to identify a reliable PICS threshold for pathological posterior compartment descent. The performance of PICS measurements in identifying pathologic anterior and middle compartment descent is comparable to established PCL measurements.
